# Functional Supramolecular of Inclusion Complex of Herbicide Fluroxypyr with HPβCD

**DOI:** 10.3390/polym10121294

**Published:** 2018-11-22

**Authors:** Shuang Gao, Chao Bie, Yanyan Liu, Tianyu Zhang, Ying Fu, Fei Ye

**Affiliations:** College of Science, Northeast Agricultural University, Harbin 150030, China; gaoshuang@neau.edu.cn (S.G.); biechao@my.com (C.B.); liuyanyan9166@126.com (Y.L.); alohagao@126.com (T.Z.); fuying@neau.edu.cn (Y.F.)

**Keywords:** HPβCD, fluroxypyr, inclusion complex, aqueous solubility, thermal stability

## Abstract

In this study, hydroxypropyl-β-cyclodextrin (HPβCD) was used to form an inclusion complex with fluroxypyr to enhance water solubility and thermal stability. The inclusion complex was prepared by a saturated solution method and characterized by FT-IR, SEM, TGA, and XRD. All results indicated that fluroxypyr successfully entered the HPβCD cavity. In addition, the study of phase solubility identifies that the water solubility of fluroxypyr was greatly improved after the formation of the inclusion complex, and TGA analysis suggested that the formation of the inclusion complex improved the thermal stability. Bioactivity assay tests showed that the inclusion complex still had strong herbicidal activity. Our research showed that HPβCD was a promising carrier for improving the properties of fluroxypyr and, thus, expanding its use in agrochemical formulations.

## 1. Introduction

**Figure 1:**
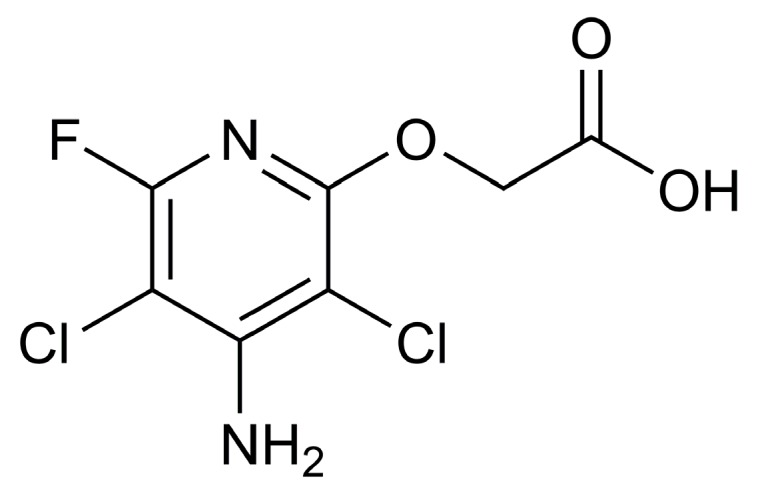
Structure of fluroxypyr.

Fluroxypyr is registered for the control of broadleaf weeds in cereals, orchards and rangeland [1]. Its chemical formula is 4-amino-3,5-dichloro-6-fluoro-2-pyridyloxyacetic acid (Figure 1). Fluroxypyr is an herbicide in the class of synthetic auxins, as well as aminopyralid, clopyralid, picloram, and triclopyr. Fluroxypyr’s mechanism of action is like that of other auxin-mimicking herbicides [2]. However, there are several factors that may limit the use of fluroxypyr. For example, it is almost insoluble in water; it is dissolved and formulated using organic solvents, and susceptible to thermal degradation. Therefore, it is important to develop an effective fluroxypyr carrier with improved solubility and stability. The formation of HPβCD inclusion complex is one of the solutions to solve the above problems [3,4].

**Figure 2:**
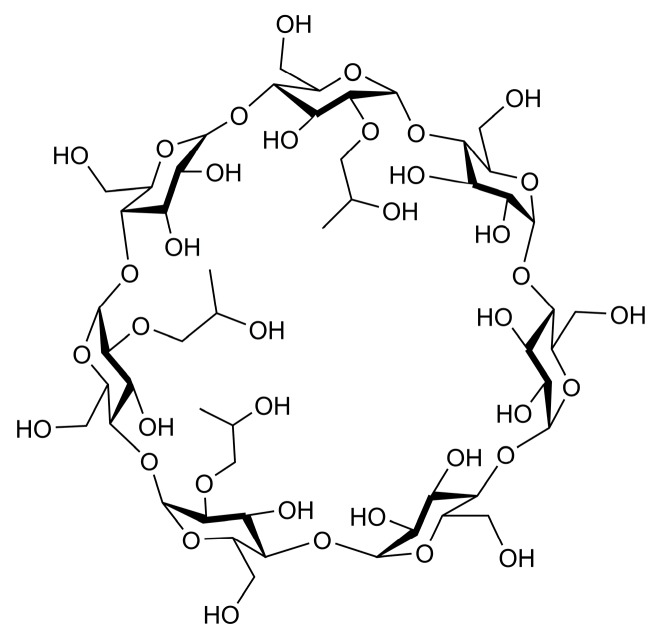
Structure of HPβCD.

HPβCD (Figure 2) is a macrocyclic carbohydrate consisting of seven D-glucose units [5]. It has a tapered structure with a hydrophilic outer surface and a relatively hydrophobic internal cavity. This enables the hydrophobic guest to be encapsulated in its cavity to form an inclusion complex. It is also environmentally friendly and inherently biodegradable.

The guest encapsulation in HPβCD may have a significant effect on its apparent aqueous solubility and susceptibility to environmental factors [6]. Studies indicated that the inclusion complex of quercetin with HPβCD exhibited modest improvement in the photostability of quercetin along with enhanced solubility [7]. Complexes between geraniol and HPβCD are considered as promising bioactive materials for the design of functional food [8]. It was also found that the plant growth regulator NAAm was susceptible to degradation by sunlight in aqueous solution. The formation of HPβCD inclusion complex mitigated the photo degradation exhibited of sunlight radiation on NAAm and improved its effective application in preparation of fruits and vegetables [9]. The inclusion complex of FeMeOH and HPβCD in a solid state was prepared by various techniques, such as coprecipitation, kneading, and freeze drying, and the cytotoxic activity of FeMeOH and its complex product with HPβCD was determined using MTT-assay on MDA-MB-231 cell line, indicating that the inclusion complex has a higher ability to inhibit cell growth than pure FeMeOH [10]. The phase solubility study was carried out by mixing an excess of vanillin with an aqueous solution containing an increased amount of HPβCD, and the results showed that the vanillin/HPβCD inclusion compound had better water solubility [11].

We know that most herbicides used in agricultural production have low water solubility and need to be dissolved with organic solvents, which seriously affects the efficacy of herbicides and increases the pollution of soil. At the same time, the thermal stability is also poor. These are all practical problems that need to be urgently solved. Unfortunately, very few of the reported studies with cyclodextrin inclusion compounds have involved herbicide cyclodextrin inclusion compounds. In our study, HPβCD was used to for the preparation of inclusion compounds with fluroxypy that encapsulated the latter molecule. The inclusion compound formed was characterized by FT-IR, SEM, and XRD, indicating that the inclusion compound has been successfully formed. TGA study shows that the inclusion compound has more thermal stability than free fluroxypyr, and the results of phase solubility study also show that the solubility of fluroxypyr has been greatly improved by forming the inclusion compound.

## 2. Materials and Methods

### 2.1. Materials

HPβCD was provided by Aladdin Reagent Co., Ltd. (Shanghai, China). Fluroxypyr was obtained from Dalian Meilun Biotech Co., Ltd. (Dalian, China). All other chemicals were obtained from Aladdin Reagent Co., Ltd. (Shanghai, China).

### 2.2. Preparation of the Inclusion Complex

**Figure 3:**
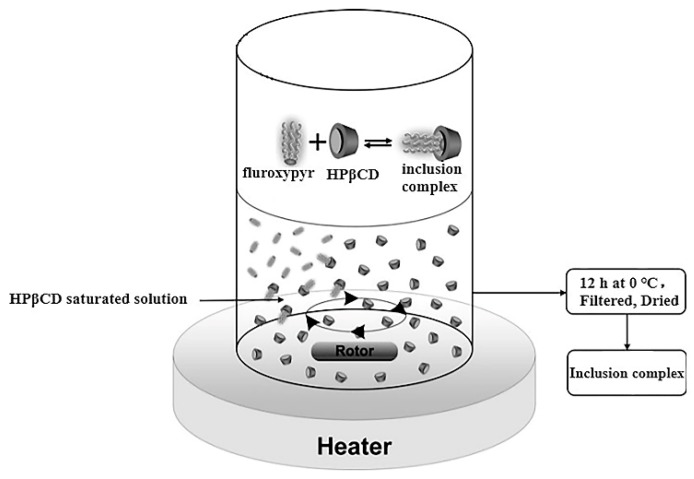
Scheme of the experimental processes to prepare the inclusion complex.

The HPβCD was dissolved in water to prepare a saturated solution. Fluroxypyr was dissolved in acetone. The fluroxypyr-acetone emulsion was gradually added to the saturated solution of HPβCD (Figure 3). After being heated at 55 °C and stirred for 2.5 h to ensure the embedding process, the mixture was incubated at 0 °C for 12 h. Then the suspension was filtered. The precipitate was washed for three times with acetone to remove residual fluroxypyr, and then dried at 60 °C for 8 h prior to weighing.

### 2.3. Preparation of Physical Mixture

The physical mixture of fluroxypyr and HPβCD was prepared in the same weight ratio as the inclusion complex. Fluroxypyr and HPβCD were mixed in a mortar and pestle for 5 min to obtain a uniform powder.

### 2.4. Characterisation of Fluroxypyr-HPβCD Inclusion Complex

#### 2.4.1. Phase Solubility Studies

Phase solubility studies were performed using the methods described by Higuchi and Connors [12]. An aqueous solution of HPβCD with concentrations ranging from 0 to 10 mM was prepared, and excess fluroxypyr was added to the above solution. The mixed solution was shaken for 48 h at 25 °C and then filtered through a 0.45 μm cellulose filter. The amount of fluroxypyr dissolved in each filtrate was determined spectrophotometrically at 211 nm using a UV-visible dual beam spectrophotometer (UV-2550, Shimadzu, Suzhou, China) with a 1 cm quartz cuvette.

The phase solubility curve was plotted to indicate the apparent water solubility of fluroxypyr as a function of the HPβCD concentration.

The association constant (*K_f_*) value was calculated by the following formula:

(1) $$K_{f}=\frac{Slope}{S_{0}\left(1-Slope\right)}$$

where *S*_0_ was the inherent water solubility of fluroxypyr in water and the *Slope* represents the slope of the phase solubility plot.

Complexation efficiency (*CE*) was calculated using the following equation:

(2) $$CE=S_{0}\times K_{f}=\frac{\left[\mathrm{HP}\beta \mathrm{CD}/\mathrm{fluroxypyr}\right]}{\left[\mathrm{HP}\beta \mathrm{CD}\right]}=\frac{Slope}{\left(1-Slope\right)}$$

where [HPβCD/fluroxypyr] was the concentration of the solubilized HPβCD/fluroxypyr inclusion complex and [HPβCD] was the concentration of the free HPβCD. By studying the solubility of the phase, the effect of forming the inclusion complex on the water solubility of the fluroxypyr could be further analyzed.

#### 2.4.2. Fourier Transform Infrared Spectroscopy (FT-IR)

FT-IR spectra were recorded in KBr using a Shimadzu 8400S FT-IR spectrometer (Shimadzu, Kyoto, Japan. Spectral smoothing and baseline correction were used. The FT-IR spectrum of HPβCD showed a wide strong peak between 3300–3400 cm^−1^, and fluroxypyr showed a sharp strong peak around 3300 cm^−1^, the FT-IR spectrum of the physical mixture was a simple superposition of HPβCD and fluroxypyr. However, all of these peaks moved to lower frequencies in the FT-IR spectrum of the inclusion complex. The differences in the positions of the peaks corresponding to fluroxypyr, HPβCD, the physical mixture, and inclusion complex could be clearly compared by FT-IR study, which provided a partial basis for the determination of the inclusion complex formation.

#### 2.4.3. Scanning Electron Microscopy (SEM)

An SU-8010 environmental scanning electron microscope system (Hitachi Company, Tokyo, Japan) was used to determine the morphological changes of HPβCD and the inclusion complex. The sample was mounted on a sample holder with an aluminum strip and sputtered with a thin gold layer under high vacuum conditions with an acceleration voltage of 12.5 kV. SEM could provide an intuitive image for us to analyze whether the inclusion complex had been successfully formed by comparing the image differences of fluroxypyr and the inclusion complex.

#### 2.4.4. Thermal Gravity Analysis (TGA)

TGA (Netzsch Company, Shanghai, China) was used to confirm the formation of the inclusion complex. Approximately 2–4 mg of sample (HPβCD, fluroxypyr, physical mixture or the inclusion complex) was placed in a flat-bottomed aluminum pan and heated at a constant rate of 10 °C min^−1^ using differential scanning under a constant stream of dry nitrogen. The calorimeter was scanned from 50–800 °C. TGA analysis could help us to check whether the thermal stability of fluroxypyr had been improved after the inclusion complex was formed.

#### 2.4.5. X-ray Diffraction (XRD)

The powder sample was packaged from the top in an X-ray holder prior to testing. The X-ray powder diffraction pattern was recorded on a Phillips X-ray diffractometer (Malvern Panalytical, Etten Leur, Netherlands) using Cu-κα (λ = 1.5406 Å) radiation, a voltage of 40 kV and a current of 30 mA. The scan rate used in the range of 5–90° was 2° min^−1^. The results of XRD analysis could be used as one of the strongest evidences for the formation of the inclusion complex.

### 2.5. Biological Activity Assay

Purslane was selected to be the indicating crop. Purslane seeds were soaked in water at a constant temperature of 30 °C for 30 min, and then placed in a Petri dish which was in an incubator at a temperature of 28 °C and a humidity of 78% for 48 h. 15 pieces of the buds with the same bud length were sowed in a 7 × 7 cm plastic bowl. After 15 days of growth under indoor lighting, fluroxypyr (0.045 mmol/m^2^) and the inclusion complex (0.045 mmol/m^2^) were sprayed onto the leaves of *Portulaca oleracea* in a 1 m^2^ area. The root length, plant height and fresh weight of the purslane seedlings were determined after 10 days. Each treatment was repeated for three times and the results were analyzed and discussed.

## 3. Results

### 3.1. Phase Solubility Studies

**Figure 4:**
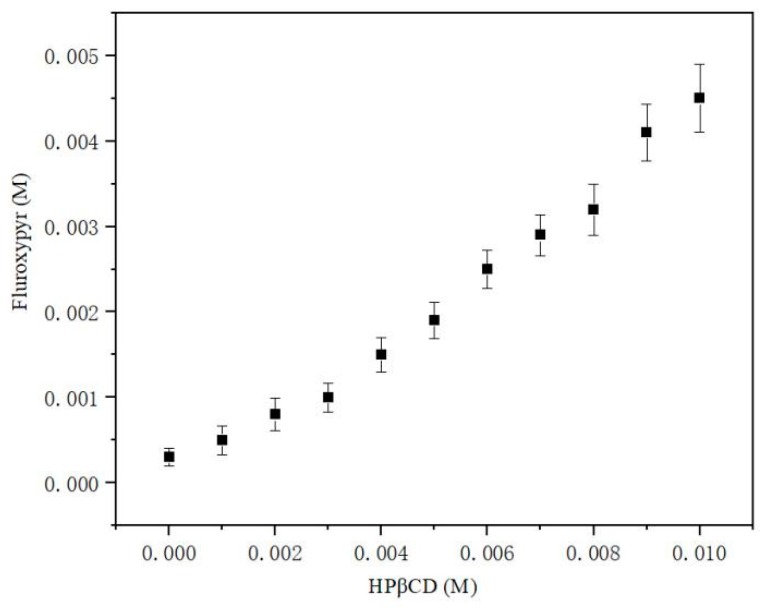
Phase solubility profiles of HPβCD/fluroxypyr inclusion complex.

The phase solubility profiles (Figure 4) showed that the aqueous solubility of fluroxypyr increased with HPβCD concentration. The phase solubility diagram obtained with HPβCD could be defined as AL-type. *K_f_* and *CE* were determined using the linear portion of the phase solubility diagram.

The *K_f_* value for HPβCD/fluroxypyr was equal to 2231 ± 223 M^−1^. This value showed that HPβCD could form stable inclusion complex with fluroxypyr. Solubility enhancement of fluroxypyr by HPβCD was shown to be related to a higher *CE* value [13]. The high *CE* value obtained in our study (0.8 ± 0.07 for HPβCD/fluroxypyr) indicated a great solubilizing potential for HPβCD towards fluroxypyr. Altogether, these results indicated that fluroxypyr and HPβCD could form the inclusion complex, and the water solubility of fluroxypyr could be significantly improved after the formation of the inclusion complex [14].

### 3.2. Fourier Transform Infrared Spectroscopy (FT-IR)

**Figure 5:**
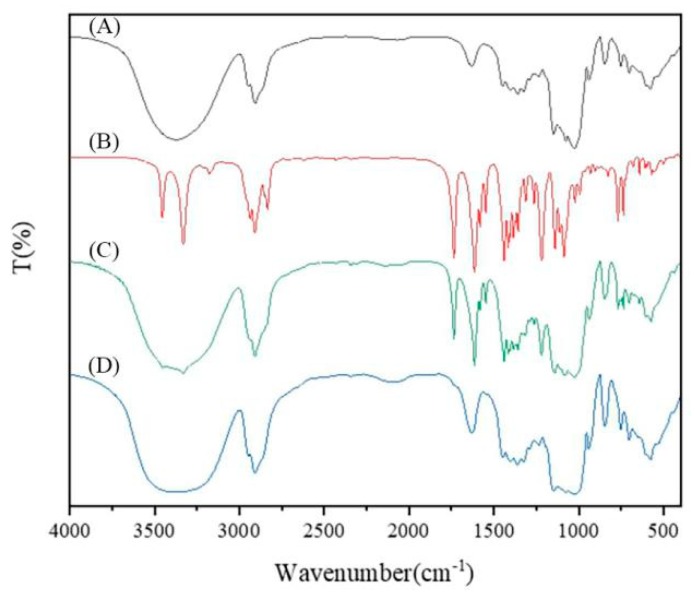
Results of Fourier transform infrared spectroscopy: (**A**) HPβCD; (**B**) fluroxypyr; (**C**) physical mixture; and (**D**) the inclusion complex.

The FT-IR spectrum of the fluroxypyr-HPβCD complex, the pure compound and the physical mixture were shown in Figure 5. The FT-IR spectrum of fluroxypyr exhibited an –NH_2_ stretching vibration at 3451 cm^−1^; an –OH stretching vibration at 3326 cm^−1^, and for a –C=O stretching vibration at 3326 cm^−1^. The FT-IR spectrum of HPβCD showed clear peaks at 3343, 1638, 1035 and 564 cm^−1^. However, all of these peaks shift to a lower frequency in the spectrum of the inclusion complex. The changes in the IR spectrum of the drug complexed with HPβCD might be due to the limitation on the vibration of the fluroxypyr molecules when molecules were encapsulated into the HPβCD cavity. This was further related to the decomposition of intermolecular hydrogen bonds in the fluroxypyr molecules and the establishment of weaker forces in complex systems such as dipole-dipole interaction, hydrophobic interaction, electrostatic attraction, and van der Waals force. Although these forces were weak, if they acted simultaneously, their binding forces were comparable to covalent bonds. In general, the force between the host and the guest molecule was the sum of the above forces. The stronger the interaction between the host and the guest molecule, the easier it was to form the inclusion complex. Furthermore, the observed decrease in the strength of the peak of fluroxypyr carbonyl might be attributed to its inclusion in the HPβCD cavity.

### 3.3. The Results of Scanning Electron Microscopy (SEM)

**Figure 6:**
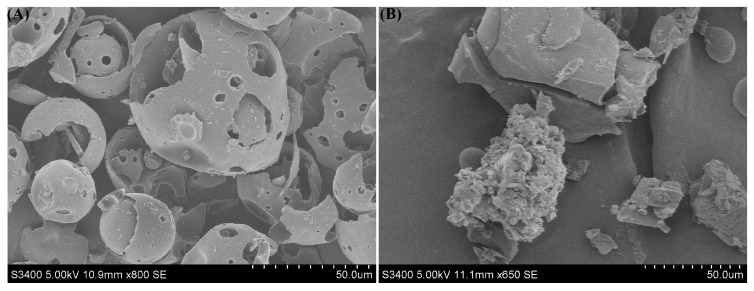
Scanning electron microscopy image of (**A**) HPβCD, and (**B**) the inclusion complex.

After the inclusion complex was formed, the crystallinity of the fluroxypyr molecules entering the HPβCD cavity might be reduced or even lost; the crystal state and spatial structure of the HPβCD molecule might also change, so that the changes could be observed by SEM. It was possible to judge whether or not the inclusion complex was formed by morphological changes. Figure 6 shows the SEM image of HPβCD and the inclusion complex. The SEM image of HPβCD was spherical or porous. Meanwhile, the inclusion complex had irregular and various block crystal structures with loose connections on the surface and lengths ranging from 5 to 10 µm. Morphological changes might be caused by the formation of the inclusion complex, which indicated that the interaction between fluroxypyr and HPβCD molecules caused a significant change in the morphology of HPβCD molecules in the inclusion complex.

### 3.4. Thermal Gravity Analysis (TGA)

**Figure 7:**
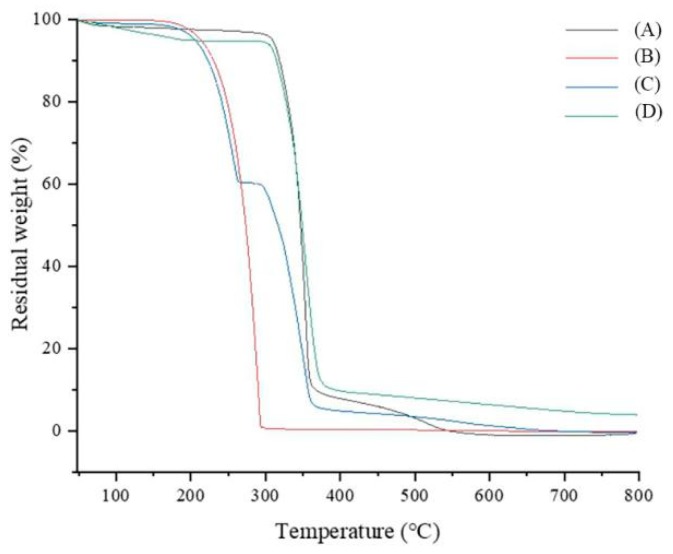
Results of thermal gravity analysis: (**A**) physical mixture; (**B**) fluroxypyr; (**C**) HPβCD; and (**D**) the inclusion complex.

TGA was employed to investigate the thermal properties of fluroxypyr [15], HPβCD, the inclusion complex and physical mixture (Figure 7).

Fluroxypyr exhibited an endothermic endothermic event at 180 °C, and the HPβCD mass loss process could be divided into two phases. In the first phase (up to 100 °C), 13.8% of the total mass was lost due to the release of water molecules. The second mass loss zone began at a temperature of 308 °C which was attributed to the decomposition of HPβCD. Regarding the TGA of the inclusion complex and physical mixture, it could be seen that the thermograms were almost the same, but the weight loss rate of the inclusion complex was slower [16]. Altogether, the inclusion complex showed better thermal stability than fluroxypyr, HPβCD and physical mixture.

### 3.5. X-ray Diffraction (XRD)

**Figure 8:**
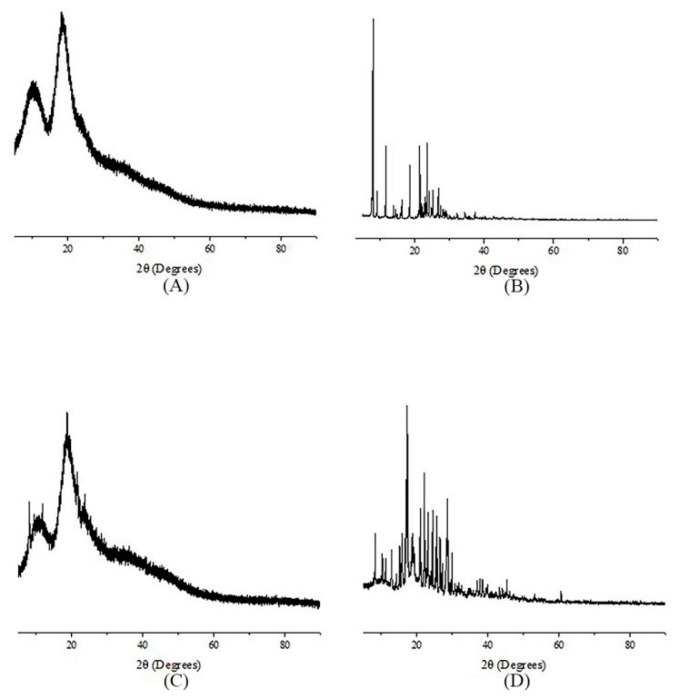
Results of powder X-ray diffractogram: (**A**) HPβCD; (**B**) fluroxypyr; (**C**) the inclusion complex; and (**D**) physical mixture.

The complexation of fluroxypyr and HPβCD was further studied by XRD. The results were shown in Figure 8. There was no obvious peak in the X-ray diffraction spectrum of pure HPβCD. In the X-ray diffraction pattern of fluroxypyr, a sharp diffraction peak was obtained, indicated the crystalline state of the drug. The same peak could be observed in the physical mixture, with lower intensity. In contrast, in the complex form, we found that the diffraction pattern was completely diffused, which indicated an amorphous structure. A lack of crystallinity was additional evidence of the inclusion complex formation [17]. This behavior suggested that there was an interaction between fluroxypyr and HPβCD [18], resulting in the formation of the fluroxypyr-HPβCD system, characterized by large diffraction peaks. These results indicated that fluroxypyr was no longer presented as a crystalline material, and that its HPβCD solid complex existed in an amorphous state. The formation of an amorphous state proved that fluroxypyr was dispersed in the molecular state of HPβCD [19,20].

### 3.6. The Result of Biological Activity Assay

The average root length of the purslane sprayed with fluroxypyr was 0.80 ± 0.10 cm, the average plant height was 3.53 ± 0.13 cm, and the average fresh weight was 0.20 ± 0.08 g; the average root length of the sprayed physical mixture was 0.76 ± 0.12 cm, the average plant height was 3.66 ± 0.11 cm, and the average fresh weight was 0.21 ± 0.07 g; the average root length of the sprayed inclusion complex was 0.70 ± 0.10 cm, the average plant height was 3.0 ± 0.08 cm, and the average fresh weight was 0.17 ± 0.05 g; the average root length of the water-sprayed purslane was 1.60 ± 0.13 cm, the average plant height was 7.23 ± 0.10 cm, and the average fresh weight was 0.47 ± 0.05 g. Table 1 shows the average data from three bioassay tests.

**Figure 9:**
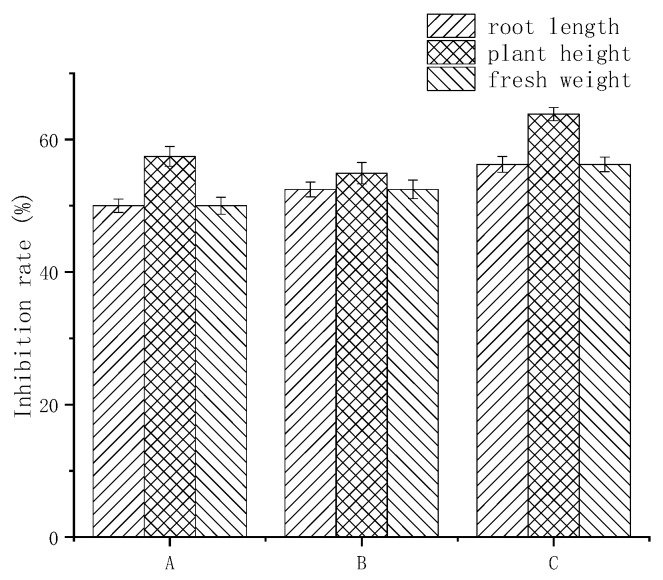
The result of biological activity assay: (**A**) fluroxypyr; (**B**) physical mixture; and (**C**) the inclusion complex.

The above results showed that the growth of purslane sprayed with fluroxypyr and with the inclusion complex was inhibited to a certain extent, indicating that the herbicidal activity of the herbicide was retained after the inclusion of the inclusion complex, and the herbicidal activity might be improved (Figure 9).

## 4. Discussion

We studied HPβCD-encapsulated fluroxypyr and confirmed by FT-IR, SEM, XRD, and other characterization methods that the inclusion complex had been formed. The FT-IR peak position of the fluroxypyr after formation of the inclusion complex, the peak intensity and peak position in the XRD pattern, and the morphology observed by SEM had great differences. The phase solubility study showed that the water solubility of fluroxypyr had been greatly improved by forming the inclusion complex. TGA analysis showed that the thermal stability of fluroxypyr had been improved to a certain extent after the formation of the inclusion complex. At the same time, the results of biological activity measurement also showed that fluroxypyr’s herbicidal activity had not been lost and had been improved to a certain extent after forming the inclusion complex. These results suggested that HPβCD complexation might be a promising strategy for improving the application of herbicides in agricultural production.

## Figures and Tables

**Table 1:** Average data from three bioassay tests.

Drugs	The Average Root Length (cm)	The Average Plant Height (cm)	The Average Fresh Weight (g)
fluroxypyr	0.80 ± 0.10 b	3.53 ± 0.13 b	0.20 ± 0.08 b
physical mixture	0.76 ± 0.12 b	3.66 ± 0.11 b	0.21 ± 0.07 b
inclusion complex	0.70 ± 0.10 b	3.00 ± 0.08 c	0.17 ± 0.05 c
water	1.60 ± 0.13 a	7.23 ± 0.10 a	0.47 ± 0.05 a

Note: Mean ± standard deviation. Values sharing same letters (a, b, c) differ non-significantly different (*p* > 0.05). The values correspond to averages of three replicates.
